# Supplementary material for: Evaluation of the Antifungal Activity of Endophytic and Rhizospheric Bacteria against Grapevine Trunk Pathogens
Source: Microorganisms. 2022 Oct 14;10(10):2035. doi: 10.3390/microorganisms10102035 (PMC9611468; doi:10.3390/microorganisms10102035)
Supplement: Supplementary file 1 [file microorganisms-10-02035-s001.zip › Table S1.pdf]

Table S1. GenBank accession numbers of strains used for phylogenetic analysis of the *Bacillus subtilis* species complex. Sequences generated on this study are highlighted in bold.

Species	Strain	16S rRNA	*gyrA*	*rpoB*	*polC*	*purH*	*groEL*
Bacillus amyloliquefaciens	ATCC 23350^T	NR_118950	FN597644:7010-9469	FN597644:122979-126560	FN597644:1728311-1732624	FN597644:668235-669773	FN597644:572972-574606
Bacillus atrophaeus	NRS-213^T	NR_116190	EF026731	EU138861	EU138723	EU138792	EU138585
Bacillus cereus	ATCC 14579^T	NR_074540	CP034551:6195-8666	CP034551:113629-117162	CP034551:139550-140494	CP034551:308734-310269	CP034551:257545-259179
Bacillus inaquosorum	NRRL B-23052^T	KT989848	GQ488737	EU138812	EU138674	EU138743	EU138536
Bacillus licheniformis	ATCC 14580^T	X68416	AE017333:6900-9368	AE017333:120755-124336	AE017333:1832492-1836808	AE017333:707082-708620	AE017333:626726-628360
Bacillus mojavensis	NRRL B-14698^T	AB021191	EU138598	EU138805	EU138667	EU138736	EU138529
Bacillus nakamurai	NRRL B-41091^T	LSAZ01000028	LSAZ01000005:218-2686	LSAZ01000009:20722-24303	LSAZ01000041:267992-272305	LSAZ01000023:39384-40922	LSAZ01000021:7656-9290
Bacillus pumilus	NRRL NRS-272^T	NR_116191	EU138655	EU138862	EU138724	EU138793	EU138586
Bacillus siamensis	KCTC 13613^T	MN176482	AJVF01000039:264-2723	KC608574	AJVF01000013:140847-144185	AJVF01000023:36481-38019	AJVF01000023:98236-99870
Bacillus sonorensis	NRRL B-23154^T	AF302118	EU138611	EU138818	EU138680	EU138749	EU138542
Bacillus spizizenii	NRRL B-14472^T	CP002183:9750-11308	EF134424	CP002183:116233-119814	CP002183:1683631-1687944	CP002183:676895-678433	CP002183:628489-630114
Bacillus subtilis	NRRL NRS-744^T	NR_116192	NC_000964:6994-9459	NC_000964:121919-125500	NC_000964:1727133-1731446	NC_000964:708594-710132	NC_000964:650234-651868
Bacillus tequilensis	NRRL B-41771^T	NR_104919	EU138625	EU138832	EU138694	EU138763	EU138556
Bacillus vallismortis	NRRL B-14890^T	AB021198	EU138601	EU138808	EU138670	EU138739	EU138532
Bacillus velezensis	NRRL B-41580^T	AY603658	EU138622	EU138829	EU138691	EU138760	EU138553
Bacillus velezensis	NRRL BD-545	-	EU138626	EU138833	EU138695	EU138764	EU138557
Bacillus velezensis	NRRL BD-568	-	EU138631	EU138838	EU138700	EU138769	EU138562
Bacillus velezensis	NRRL BD-621	-	EU138650	EU138857	EU138719	EU138788	EU138581
Bacillus velezensis	**CE100**	**OP550064**	**OP561954**	**OP561962**	**OP561970**	**OP561978**	**OP561986**
Bacillus velezensis	**UCD10598**	**OP550065**	**OP561955**	**OP561963**	**OP561971**	**OP561979**	**OP561987**
Bacillus velezensis	**UCD10599**	**OP550066**	**OP561956**	**OP561964**	**OP561972**	**OP561980**	**OP561988**
Bacillus velezensis	**UCD10600**	**OP550067**	**OP561957**	**OP561965**	**OP561973**	**OP561981**	**OP561989**
Bacillus velezensis	**UCD10607**	**OP550068**	**OP561958**	**OP561966**	**OP561974**	**OP561982**	**OP561990**
Bacillus velezensis	**UCD10613**	**OP550069**	**OP561959**	**OP561967**	**OP561975**	**OP561983**	**OP561991**
Bacillus velezensis	**UCD10614**	**OP550070**	**OP561960**	**OP561968**	**OP561976**	**OP561984**	**OP561992**
Bacillus velezensis	**UCD10631**	**OP550071**	**OP561961**	**OP561969**	**OP561977**	**OP561985**	**OP561993**
