# Supplementary material for: Evaluation of the Antifungal Activity of Endophytic and Rhizospheric Bacteria against Grapevine Trunk Pathogens
Source: Microorganisms. 2022 Oct 14;10(10):2035. doi: 10.3390/microorganisms10102035 (PMC9611468; doi:10.3390/microorganisms10102035)
Supplement: Supplementary file 1 [file microorganisms-10-02035-s001.zip › Table S2.pdf]

Table S2. GenBank accession numbers of strains used for phylogenetic analyses of *Pseudomonas* spp. and *Serratia* spp. Sequences generated on this study are highlighted in bold.

Species	Strain	16S rRNA
Pseudomonas aeruginosa	LMG 1242^T	Z76651
Pseudomonas brassicacearum	DBK11^T	NR_024950
Pseudomonas chlororaphis subsp. *aurantiaca*	NCIB 10068^T	DQ682655
Pseudomonas chlororaphis subsp. *aureofaciens*	DSM 6698^T	AB680099
Pseudomonas chlororaphis subsp. *chlororaphis*	DSM 50083^T	KX186940
Pseudomonas chlororaphis subsp. *piscium*	JF3835^T	FJ168539
Pseudomonas chlororaphis	**UCD10653**	**OP550072**
Pseudomonas chlororaphis	**UCD10746**	**OP550078**
Pseudomonas chlororaphis	**UCD10748**	**OP550079**
Pseudomonas chlororaphis	**UCD10757**	**OP550080**
Pseudomonas chlororaphis	**UCD10763**	**OP550083**
Pseudomonas donghuensis	HYS^T	NR_136501
Pseudomonas donghuensis	**UCD10759**	**OP550081**
Pseudomonas granadensis	F-278,770^T	HG764746
Pseudomonas granadensis	**UCD10729**	**OP550074**
Pseudomonas koreensis	Ps 9-14^T	NR_025228
Pseudomonas koreensis	**UCD10732**	**OP550075**
Pseudomonas cf. *koreensis*	**UCD10666**	**OP550073**
Pseudomonas cf. *koreensis*	**UCD10738**	**OP550076**
Pseudomonas cf. *koreensis*	**UCD10739**	**OP550077**
Pseudomonas kribbensis	46-2^T	KT321658
Pseudomonas monteilii	CIP 104883^T	AF064458
Pseudomonas plecoglossicida	NBRC 103162^T	BBIV01000080
Pseudomonas cf. *plecoglossicida*	**UCD10762**	**OP550082**
Pseudomonas putida	NBRC 14164^T	NR_113651
Pseudomonas reinekei	MT1^T	AM293565
Pseudomonas taiwanensis	BCRC 17751^T	EU103629
Pseudomonas vranovensis	CCM 7279^T	AY970951
Serratia entomophila	DSM 12358^T	NR_025338
Serratia ficaria	DSM 4569^T	A5233428
Serratia marcescens	DSM 30121	AJ233431
Serratia odorifera	DSM 4582^T	A5233432
Serratia plymuthica	DSM 4540^T	NR_114579
Serratia plymuthica	DSM 49	AF286871
Serratia plymuthica	CKQ9	OP035846
Serratia plymuthica	CTB4	OP102591
Serratia plymuthica	KAR18	KR054980
Serratia plymuthica	PR	ON337524
Serratia plymuthica	**UCD10719**	**OP550084**
Serratia plymuthica	**UCD10756**	**OP550085**
Serratia proteamaculans	DSM 4543^T	NR_025341
Serratia quinivorans	DSM 4597^T	NR_037112
